# An in vitro study elucidating the synergistic effects of aqueous cinnamon extract and an anti-TNF-α biotherapeutic: implications for a complementary and alternative therapy for non-responders

**DOI:** 10.1186/s12906-024-04438-w

**Published:** 2024-03-23

**Authors:** Shubrata Khedkar, Minhaj Ahmad Khan

**Affiliations:** Department of Biochemistry, School of Bioengineering and Biosciences, Lovely Professional University, Jalandhar, 144411 Punjab India

**Keywords:** Cinnamon, Aqueous extract, Infliximab, Inflammation, CAM

## Abstract

**Background:**

Tumor necrosis factor-alpha (TNF-α) is a critical pro-inflammatory cytokine, and its abnormal production is associated with several immune mediated inflammatory diseases (IMID). Biological anti-TNF-α therapy includes treatment with monoclonal antibodies such as infliximab which have proven successful and are well-tolerated in most patients. Unfortunately, some patients may not respond to therapy (primary non-responders) or may lose sensitivity to the biological agent over time (early and late secondary non-responders). Natural products can reduce inflammation and act synergistically with small molecules or biologics, although evidence remains limited. This study aimed to investigate whether complementary and alternative medicine (CAM) could play a role in infliximab non-responders. Reportedly, cinnamon can help manage chronic inflammatory conditions owing to its anti-inflammatory properties.

**Methods:**

We studied the synergistic effects of cinnamon and infliximab in vitro using a two-step approach. First, we investigated whether cinnamon and infliximab act synergistically. Second, we selected conditions that supported statistically significant synergy with infliximab and studied the mRNA expression of several genes involved in non-response to infliximab. We used aqueous cinnamon extract (aCE) from *Cinnamomum cassia*, *Cinnamomum zeylanicum*, and *Cinnamomum loureiroi* and bioactive trans-cinnamaldehyde (TCA), cinnamic acid (CA), and eugenol to study the synergy between infliximab and aCE/bioactive compounds using bioassays in fibroblast (L929) and monocytic (U937) cell lines, followed by qPCR for molecular-level insights. TCA, *C. cassia* aCE, and *C. zeylanicum* aCE demonstrated a dose-dependent synergistic effect with infliximab. Moreover, we saw differential gene expression for adhesion molecules, apoptotic factors, signaling molecules, and matrix remodelers in presence and absence of aCE/bioactives.

**Results:**

CAM supplementation was most effective with *C. cassia* aCE, where a synergistic effect was observed for all the tested genes specifically for MMP-1, BcL-xL, Bax and JAK2, followed by TCA, which affected most of the tested genes except TLR-2, MMP1, MMP3, TIMP-1, and BAX, and *C. zeylanicum* aCE, which did not affect ICAM-1, VCAM-1, TLR-2, TLR-4, MMP1, MMP3, TIMP-1, and STAT3.

**Conclusion:**

In conclusion, cinnamon acted synergistically with infliximab to mitigate inflammation when used as an extract. Purified bioactive TCA also showed synergistic activity. Thus, aCE, or cinnamon bioactive may be used as a CAM to improve patients’ quality of life.

**Supplementary Information:**

The online version contains supplementary material available at 10.1186/s12906-024-04438-w.

## Introduction

The immune system protects the host from various disease-causing agents, and one of the mechanisms by which it brings out the effect is by initiation of a protective response in the form of inflammation. However, autoimmune diseases such as Crohn's disease (CD), ulcerative colitis (UC), and rheumatoid arthritis (RA) set in when the host immune system turns against itself [1]. Approximately 5 − 8% of the world’s population is affected by autoimmune diseases [1], and approximately 80 autoimmune diseases are known so far [2]. Several autoimmune diseases associated with chronic unresolved inflammation fall under the umbrella of immune-mediated inflammatory diseases (IMIDs) [3] and share common inflammatory pathways [4]. Non-steroidal anti-inflammatory drugs, analgesics, and glucocorticoids represent the conventional treatment options, whereas biological agents represent a new line of therapy that has proven to be successful in managing IMIDs, such as ankylosing spondylitis (AS), Rheumatoid arthritis (RA), and psoriatic arthritis (PA) [2].

Tumor necrosis factor-alpha (TNF-α) is a critical pro-inflammatory cytokine, and its abnormal production is associated with several IMIDs. Biological anti-TNF-α therapy includes treatment with monoclonal antibodies such as infliximab and adalimumab and Fc fusion proteins such as etanercept [5]. These anti-TNF-α biological agents bind and neutralize TNF-α, which then cannot bind to its cognate receptor. Biological therapies have been successful and well-tolerated in most patients [6]. Regulatory authorities recommend anti-TNF-α monoclonal antibody therapy over other conventional treatments due to its targeted mode of action. Although the cost associated with these biologics is high, the development and availability of biosimilars have benefited patients considerably, especially in developing countries [7].

Unfortunately, some patients may not respond to therapy (primary non-responders) or may lose sensitivity to the biological agent over time (early and late secondary non-responders) [8]. In primary non-responders, the failure to respond to treatment is associated with several factors, including the presence of genetic variants (single nucleotide polymorphisms) [9] and differential expression [10] of genes that play critical roles in inflammatory pathways. One possible reason for the secondary non-response includes the formation of anti-drug antibodies. However, despite the development of anti-drug antibodies, patients continue to show a therapeutic response to the same biological agents [6]. In non-responders, increasing the dose, switching, or swapping of different biologic agents have been reported. Clinical benefit has been observed in 50 − 70% of patients with increased amounts, while approximately 50% of patients have benefitted after switching the biologic. Some studies have also shown that combination therapy with immunosuppressants (initiated after non-response to higher doses of biologics) in non-responders was promising in half of the tested patients [11]. Immunosuppressants and other small-molecule agents are associated with toxicity in patients. Therefore, in addition to the ongoing approaches, there is an unmet requirement of identifying non-toxic molecules that work synergistically with anti-TNF-α monoclonal antibodies to initiate and sustain appropriate responses in biological non-responders to anti-TNF-α therapy.

Several reports have indicated that dietary products, including spices, are involved in the management of IMIDs [12, 13]. Cinnamon (*Cinnamomum* spp.) has been widely used as a spice and has been reported to possess anti-inflammatory properties [14, 15] and several other beneficial effects, such as anticancer and antimicrobial effects [16, 17]. Some reports have indicated that cinnamon can decrease the levels of low-density lipoprotein, total cholesterol, and serum triglycerides in diabetic patients [18]. Hall et al. [19] published a study on the use of natural health products (NHP) in treating rheumatological conditions in which he reported that ~ 36% of the study participants (out of 1,063) used NHP. For some NHP users (42%), the NHP recommendations came from the physicians, indicating the acceptance of these complementary and alternative therapies by patients and physicians alike, albeit to a limited extent. Natural products, including cinnamon, have been reported to alter several signaling pathways [20]. *Cinnamomum verum* essential oil has been reported to decrease the expression of Toll-like receptor-4 (TLR-4) in a dextran sodium sulfate-induced model of colitis and has been suggested to be effective in treating inflammatory bowel disease (IBD). Similar results have been observed for curcumin and many other natural products [21]. In another study, aqueous cinnamon extract (aCE) partially controlled bowel symptoms by downregulating tryptophan hydroxylase 1 in a model of irritable bowel syndrome [22]. It has also been reported that cinnamon extract acts as a pro-apoptotic agent, downregulating BCL2, BcL-xL and Survivin [15]. A recently published single-center study explored the adjuvant effects of purple corn supplementation on IBD remission. The results showed that purple corn supplementation reduced the expression of inflammatory biomarkers in patients with CD undergoing infliximab treatment and improved the infliximab response. However, this effect was not observed in patients with UC [23]. Unfortunately, such studies are rare.

To address this gap in the literature, we aimed to study the synergistic effects of cinnamon, specifically the commonly used species *Cinnamomum zeylanicum, Cinnamomum cassia,* and *Cinnamomum loureiroi*, with infliximab. In particular, we focused on the aCE because of its simple method of preparation. For this study, we selected infliximab, as it is widely used in biotherapy for IMIDs [2]. We studied the effects of the extracts from multiple cinnamon species on the potency of infliximab in an in vitro assay using fibroblast and monocyte cell lines, as both these cell types are actively involved in inflammatory pathogenesis. The cinnamon species that showed synergistic activity in the in vitro assay were further used to examine changes in the expression of various genes that are known to play critical roles in exacerbating inflammation in non-responders. In this study we show that cinnamon aCE can work synergistically with infliximab to enhance its effectiveness and paves a way forward for in vivo studies. Eventually, aCE can be used as a complementary and alternative medicine (CAM) to improve the quality of life of patients.

## Materials and methods

### Reagents and consumables

Cinnamon powder was purchased from a local distributor. *C. cassia* was from Carmel Organics, Barukheda, India; *C. loureiroi* was from Kirkland, D.C, USA; *C. zeylanicum* was from Sorich Organics, Noida, India. Infliximab was purchased from a local Pharmacy in India. Cell culture reagents were purchased from Corning (NY, USA). All other reagents were purchased from the vendors as listed. Caspase3/7 glo reagent (G8090, Promega Madison, WI, USA), TNF-α (AB155699, Abcam, Waltham, MA, USA), trans-cinnamaldehyde (TCA; C80687, Sigma, Burlington, MA, USA), cinnamic acid (CA; 8.00235, Sigma), eugenol (E51791, Sigma), actinomycin D (A9415, Sigma), trypsin–EDTA (25,200–056, Gibco, Billings, MT, USA), phosphate-buffered saline (PBS), pH 7.2 (20,012–027), and Alamar Blue (DAL1100, Invitrogen, Waltham, MA, USA).

### Preparation of aCE

Ground cinnamon (*C. zeylanicum, C. cassia,* and *C. loureiroi*) bark powder was dissolved in water (70 °C for 1 h) to obtain 100 mg/mL extracts. All extracts were centrifuged (12,000 rpm for 10 min) to remove the insoluble components. The supernatant was filtered (0.4 µm pore size, HNWP04700, Merck Millipore Burlington, USA), aliquoted, and stored at -80 °C.

### Cell lines and cultures

The L929 (ATCC CCL-1) and U937 (ATCC CRL-1593.2) cell lines were purchased from the American Type Culture Collection (Manassas, VA, USA). The L929 cell line was cultured under aseptic conditions in Eagle’s minimum essential media (EMEM; M4655, Sigma), and the U937 cell line was cultured under aseptic conditions in Roswell Park Memorial Institute-1640 medium (RPMI-1640; M8758, Sigma). Both media were supplemented with 10% (v/v) fetal bovine serum (FBS; 10,082–147, Gibco) and 1% (v/v) penicillin/streptomycin (P4333, Sigma-Aldrich). The cell cultures were maintained in a 37 °C incubator at 95% humidity and in the presence of 5% CO_2_.

### L929 and U937 cell viability assay

The L929 cells were cultured in a 75 cm^2^ flask in EMEM supplemented with 10% FBS. The assay medium contained 3% FBS in EMEM. The L929 cell suspension was prepared in assay media by trypsinizing the cell monolayer. Fifty microliters of 0.75 × 10^6^ cells/mL (37,500 cells/well) were plated in each well of the 96-well plates. aCE was serially diluted to eight different concentrations ranging from 33.3 to 0.26 mg/mL (final concentrations). 50 µL of aCE and actinomycin D was added to treated and actinomycin control wells. Control wells without aCE or actinomycin D were also established.

The U937 cells were cultured in RPMI-1640 medium supplemented with 10% FBS. The assay medium contained 2% FBS. In total, 25 µL of 1.2 × 10^6^ cells/mL (30,000 cells per/well) was plated in each well of the 96-well plates. aCE was serially diluted to eight different concentrations ranging from 33.3 to 0.26 mg/mL (final concentrations). 25 µL of aCE was added to the treated wells. Control wells without aCE were also established.

The plates were incubated at 37 °C in the presence of 5% CO_2_ for 18 − 20 h, followed by the addition of 20 µL Alamar Blue to the wells. The plates were further incubated for 6 − 7 h at 37 °C in the presence of 5%. CO_2_. The color change of alamar blue reagent was measured using a Synergy microplate reader (BioTek, Winooski, VT, U.S.) at 570 nm and 600 nm. The average absorbance of the cell culture medium alone (background) at 600 nm was subtracted from that at 570 nm. The background-subtracted absorbance at 570 nm was plotted against the concentration of the test compound. Viability assays with TCA, cinnamic acid (CA), and eugenol were performed in a manner similar to that described above (the concentration range was 1–0.65 mg/mL).

### aCE-infliximab synergy experiments

The L929 cell suspension was prepared by trypsinizing the cell monolayers. In total, 25 μL of the cell suspension (1.5 × 10^6^ cells/mL or 37,500 cells/well) was added to each untreated, treated, TNF-α control and cell control labelled wells in a transparent 96-well microplate plate and incubated for 20 min at 37 °C in an atmosphere of 5% CO_2_. Twenty-five microliters of aCE or bioactives were added to the cells (labelled treated wells) to final concentrations of 150, 50, 25, and 10 µg/mL or 40, 32, 16, 8, and 4 µg/mL (in duplicates), respectively. Each set was incubated for 15, 30, or 60 min. Twenty-five microliters of the assay medium were added to the control wells (cell control, TNF-α control) and untreated wells in the assay plate. Simultaneously, 100 μL of eight different concentrations of infliximab (30 ng to 0.23 ng/mL, final concentrations) were added to the wells marked as treated and untreated on separate neutralization plate. One hundred microliters of TNF-α (20 IU/mL, final concentration) was added to each treated, untreated, and TNF-α control well and mixed five times (this plate did not contain cells, only infliximab and TNFα in 1:1 volume ratio for neutralization) and incubated for 1 h at 37 °C. One hundred microliters of the assay media were added to the TNF-α control wells and 200 μL to the cell control wells. After 15, 30, or 60 min of incubation of aCE with cells, 50 μL of the neutralization mixture was transferred to the wells in the assay plate marked as treated and untreated. Fifty microliters of 4 μg/mL actinomycin D were added to the treated, untreated, and TNF-α control wells, and 100 μL assay medium was added to the control wells. The cell plates were incubated at 37 °C in the presence of 5% CO_2_ for 18 − 20 h, followed by the addition of 20 µL alamar blue to the wells and further incubation for 6 − 7 h at 37 °C in an atmosphere of 5%. CO_2_. The color change was read using a Synergy microplate reader (BioTek) at 570 nm and 600 nm. The average absorbance value of the cell culture medium alone (background) at 600 nm was subtracted from that of the experimental wells at 570 nm. The background-subtracted absorbance at 570 nm was plotted against the infliximab concentration to determine the shift in the half maximal effective concentration (EC50) in the presence of aCE. Similar study was carried out for bioactives (15 min incubation).

The U937 cell suspension was prepared by harvesting the cells, and then centrifuging at 125 rcf for 5 min, resuspending, and counting them in the assay medium. In total, 12.5 μL of the cell suspension (2.4 × 10^6^ cells/mL or 30,000 cells/well) was added to each untreated, treated, TNF-α control and cell control wells in an opaque white 96-well microplate plate (assay plate) and incubated for 10 min at 37 °C in an atmosphere of 5% CO_2_. aCE (12.5 μL) was added to the cells (labelled treated wells) to final concentrations of 150, 50, 25, and 10 µg/mL, or 12.5 μL bioactives were added to cells to final concentrations of 40, 32, 16, 8, and 4 µg/mL (in duplicates). Each set was incubated for 15 min. The assay medium (12.5 μL) was added to the control wells (cell control, TNF-α control), and untreated wells in the assay plate. Simultaneously, 50 μL of eight different concentrations of infliximab (50 ng to 1.14 ng/mL, final concentration) were added to the wells labelled as treated and untreated on a separate neutralization plate. Fifty microliters of TNF-α (20 IU/mL, final concentration) was added to each treated, untreated, and TNF-α control wells and mixed five times (this plate did not contain cells, only infliximab and TNFα in 1:1 volume ratio for neutralization) and incubated for 1 h at 37 °C. Fifty microliters of the assay media were added to the TNF-α control wells and 100 μL to the cell control wells. After incubation, 25 μL of the mixture was transferred to the wells marked as treated and untreated in the assay plate, and the plate was incubated for 2.5 h at 37 °C in the presence of 5% CO_2_. Fifty microliters of the caspase 3/7 glo reagent were added to the treated, untreated, TNF-α control and cell control wells, and the plates were further incubated at 30 min at room temperature before taking the reading. Luminescence was measured using a Synergy microplate reader (BioTek).

### Gene expression assay

L929 cells were added to 6-well plates (1 × 10^6^ cells per well in 600 µL) in EMEM + 10% FBS (v/v) and incubated for ~ 18 h under sterile conditions. The spent medium was discarded, and the cells were rinsed with EMEM (200 µL/well). In a separate plate, infliximab EC100 (30 ng/mL, final concentration) was mixed with TNF-α (20 IU/mL, final concentration) and incubated for 1 h at 37 °C. aCE samples and concentrations which showed significant synergy (*p*-value < 0.05) and reproducibility across days in the synergy experiments were selected for gene expression assays. The cells were preincubated with 300 µL of 50 and 25 µg/mL aCE (final concentrations) or 40 and 32 µg/mL (final concentrations) of bioactive for 15 min, followed by the addition of an equal volume of the neutralized TNF-α + infliximab (EC100 mix) and further incubated for 30, 60, or 120 min. Cell control and infliximab control wells (EC100 mix) were also set up. At each time point, the cells were harvested and used for RNA extraction and cDNA preparation. 120 min time point was selected based on data reproducibility. Each sample was analyzed in duplicates.

A U937 cell suspension was prepared in the assay medium and added to 6-well plates (1 × 10^6^ cells per well in 600 µL). In a separate plate, infliximab EC100 (50 ng/mL, final concentration) was mixed with TNF-α (20 IU/mL, final concentration) and incubated for 1 h at 37 °C. aCE samples and concentrations with a significant *p*-value  in the synergy experiments were selected for gene expression assays. Cells were preincubated with 300 µL of 50 and 25 µg/mL (final concentrations) aCE or 40 and 32 µg/mL (final concentrations) bioactive for 15 min, followed by the addition of 300 µL neutralized TNF-α + infliximab (EC100 mix) and further incubated for 120 min. Cell control and infliximab control (EC100 mix) wells were also set up. At each time point, the cells were harvested and used for RNA extraction and cDNA preparation. Each sample was analyzed in duplicate.

### RNA extraction and quantitative reverse transcription-polymerase chain reaction (qRT-PCR)

Total RNA from treated, untreated, and control samples was extracted using the RNeasy mini kit (74,101, Qiagen, Hilder, Germany) according to the manufacturer's instructions, and the concentration was measured using Take3 low-volume plates on a Synergy microplate reader (BioTek). cDNA was prepared using Protoscript II (E6560, NEB, Ipswich, MA, USA) per the product datasheet. qRT-PCR was performed using the PowerUp™ SYBR™ Green master mix (A25742, Thermo Fisher Scientific, Waltham, MA, USA) on an ABI 7500 real-time PCR system (Applied Biosystems, Waltham, MA, USA). The conditions used were initial denaturation for 10 min at 95 °C; primer annealing for 15 s at 95 °C, and elongation for 60 s at 60 °C for 40 cycles. A melting curve analysis was also performed where primer specificity was confirmed by the presence of a single peak at melting temperature. Housekeeping genes for data normalization were selected based on Norm Finder. The decrease or increase in TNF-α-induced gene expression was determined in the presence of infliximab, aCE, or bioactive compounds. Changes in gene expression were calculated in terms of fold induction with respect to the untreated cell control using the 2-∆∆CT method. The level of gene expression in TNF-α-treated controls was considered 100%. An increase or decrease in expression after infliximab treatment was the second level of control. The effects of aCE or bioactive treatment in the presence of infliximab + TNFα (EC100) were considered significant when the expression levels of various genes differed significantly from those after the infliximab (EC100) treatment alone. The primers used in this study are listed in Supplementary Table S1.

### Statistical analysis

All experiments were performed at least three times unless stated otherwise. All samples were analyzed in duplicates. Means of more than two groups were compared using one-way analysis of variance (ANOVA) with the Kruskal–Wallis test. Statistical significance was set at *p* < 0.05. Data was analyzed using the GraphPad Prism software (GraphPad Software, Inc., San Diego, CA, USA).

## Results

### Effect of aCE on cell viability

**Fig. 1:**
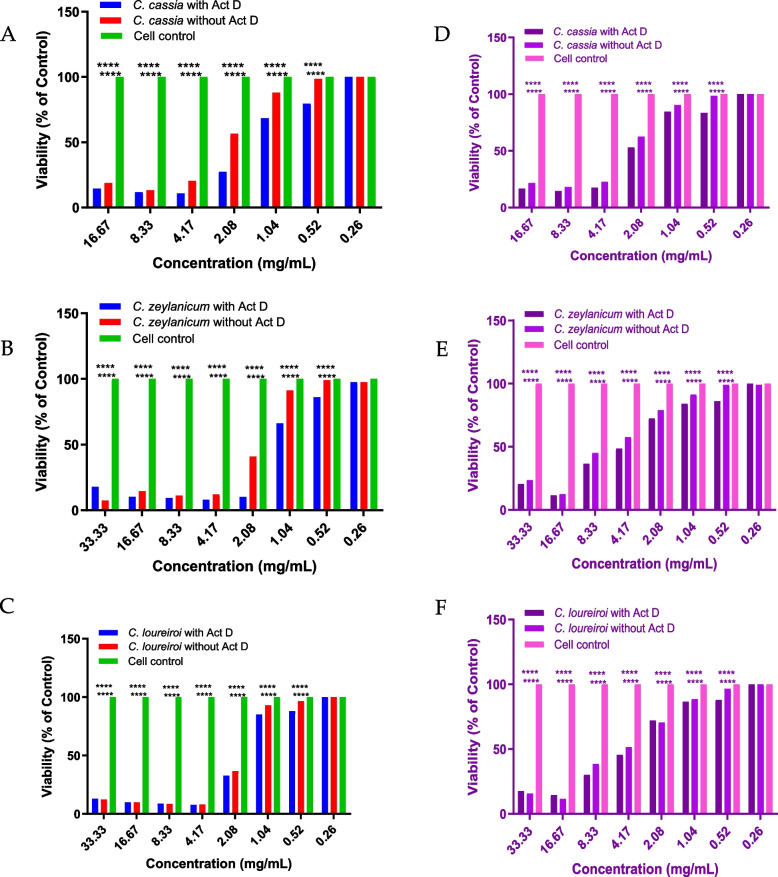
Cell viability after treatment with aCE. The L929 cells were treated with aCE (33.3 to 0.26 mg/mL, final concentrations) of *Cinnamomum cassia* (**A**), *Cinnamomum zeylanicum* (**B**), and *Cinnamomum loureiroi* (**C**) for 24 h, and viability was determined using the redox reagent, Alamar Blue. Experiments were performed thrice, and the data represent mean values. aCE, aqueous cinnamon extract. The U937 cells were treated with aCE (33.3 to 0.26 mg/mL, final concentrations) of *Cinnamomum cassia* (**D**), *Cinnamomum zeylanicum* (**E**), and *Cinnamomum loureiroi* (**F**) for 24 h, and viability was determined using the redox reagent, Alamar Blue. Experiments were performed thrice and mean of the data was plotted. aCE, aqueous cinnamon extract

We evaluated the synergistic effects of aCE with the biotherapeutic, infliximab. First, we first determined the concentration of cinnamon that was non-toxic to the cells using Alamar Blue for detecting viability. As actinomycin D was required for the L929-based synergy experiments, cell death in the presence and absence of actinomycin D was also tested. As shown in Fig. 1(A, B and C), a dose-dependent decrease in cell viability was observed for all aCE extracts with increasing concentrations. Similar results were observed in a study on the U937 cell line (Fig. 1D, E and F). For *C. cassia* aCE highest concentration was interfering with the reading hence was excluded from the analysis. Concentrations below 260 mg/mL were non-toxic to the cells for all the tested aCE. The cinnamon bioactive compounds were non-toxic at concentrations below 100 µg/mL (data not shown). Accordingly, for the synergy experiments, four concentrations of aCE (150, 50, 25, and 10 µg/mL) and five concentrations of cinnamon bioactive compounds (40, 32, 16, 8, and 4 µg/mL) were considered safe.

### Synergy experiments with aCE in the L929 cell line

**Fig. 2:**
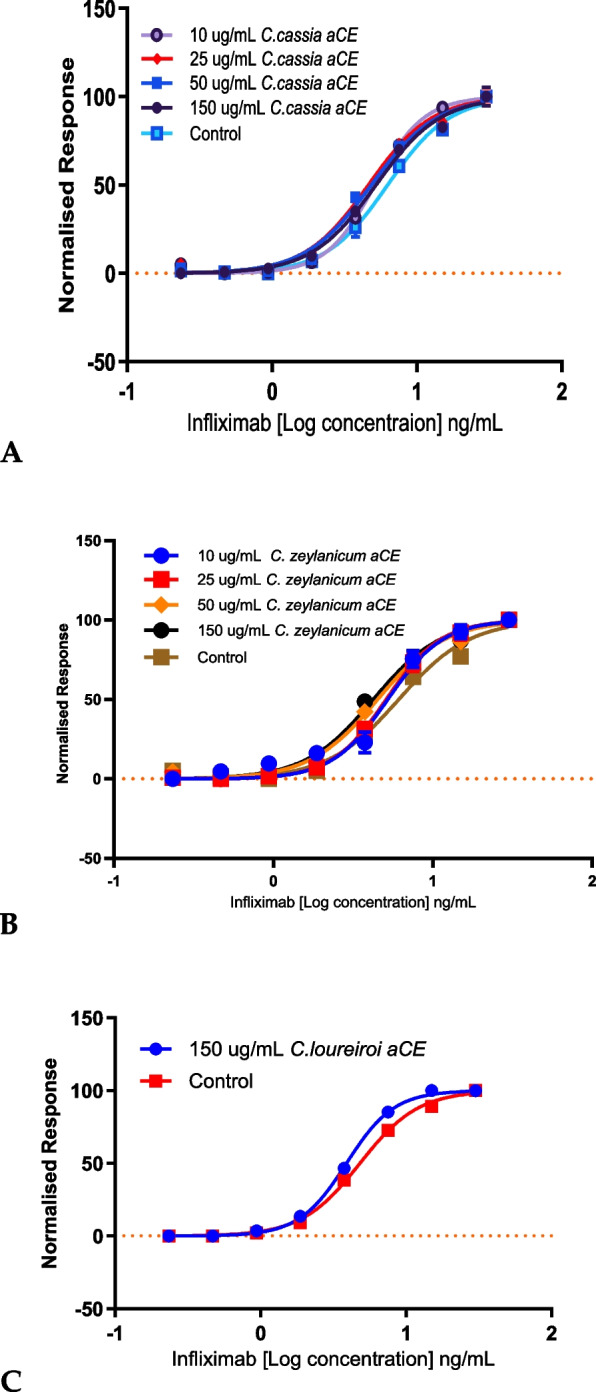
Synergy experiments with aCE at 15 min preincubation in L929 cells. All aqueous extracts showed synergy albeit to varying degrees. All tested concentrations of *C. cassia* (**A**) and *C. zeylanicum* (**B**) showed synergistic effects at 15 min, while *C. loureiroi* (**C**) showed a synergistic effect at the highest tested concentration and at 30 min only. The *p*-values for *C. cassia* (Table 1), *C. zeylanicum* (Table 2), and *C. loureiroi* (Table 3) have been summarized

The aCE of *C. cassia* showed a significant synergistic (*p* < 0.05) effect with infliximab (Fig. 2A) when preincubated for 15 min before the addition of various doses of neutralized infliximab and TNF-α solution**.** This effect was observed at all the tested concentrations of aCE (Table 1). The EC50 of infliximab in the presence of aCE was lower than that in the absence of aCE, indicating synergistic activity. At 30 min of preincubation, the effect was observed only at 150 and 50 µg/mL, and no synergy was observed at the 60 min preincubation time point (data not shown). Moreover, the data obtained at 60 min showed large variations within replicates and changes in the slope of the dose–response curve.

**Table 1:** Changes in the EC50 and corresponding *p*-values after the *Cinnamomum cassia* aCE synergy experiment in the L929 cell line

aCE concentration	Infliximab EC50 [ng/mL]	*p*-values
Control	6.262	NA
150 µg/mL	5.203	0.0002
50 µg/mL	4.828	0.0002
25 µg/mL	4.618	< 0.0001
10 µg/mL	5.115	< 0.0001

*aCE* Aqueous cinnamon extract, EC50, half maximal effective concentration

As observed with *C. cassia* aCE, *C. zeylanicum* aCE also showed significant synergy (*p* < 0.05) at 15 min and at all concentrations (Fig. 2B and Table 2). No synergy was observed at any other time points at any of the tested concentrations.

**Table 2:** Changes in the EC50 and corresponding *p*-values after the *Cinnamomum zeylanicum* aCE synergy experiment in the L929 cell line

aCE concentration	Infliximab EC50 [ng/mL]	*p*- values
Control	6.117	NA
150 µg/mL	4.237	< 0.0001
50 µg/mL	4.507	< 0.0001
25 µg/mL	5.103	< 0.0001
10 µg/mL	5.219	< 0.0001

The *C. loureiroi* aCE was the only extract that showed significant synergy (*p* < 0.05) after 30 min of preincubation. The synergy was observed only at the highest tested aCE concentration (150 µg/mL) (Fig. 2C and Table 3).

**Table 3:** Changes in the EC50 and corresponding *p*-values after the *Cinnamomum loureiroi* aCE synergy experiment in the L929 cell line

aCE concentration	Infliximab EC50 [ng/mL]	*p*-values
Control	4.815	NA
150 µg/mL	3.892	0.0343

### Synergy experiments with aCE in the U937 cell line

**Fig. 3:**
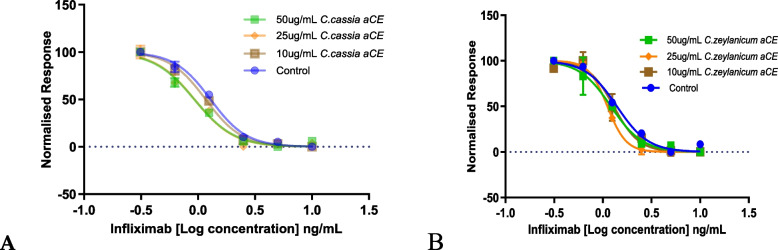
Synergy experiments with aCE at 15 min preincubation in the U937 cell line. *C. cassia* (**A**) and *C. zeylanicum* (**B**) showed synergistic effects with three tested concentrations at 15 min. The *p*-values for *C. cassia* (Table 1) and *C. zeylanicum* (Table 2) have been summarized

The aCE of *C. cassia* showed a significant synergistic (*p* < 0.05) effect with infliximab (Fig. 3A and Table 4) when preincubated for 15 min before the addition of various doses of neutralized infliximab and TNF-α solution**.** For the U937 cell line, 150 µg/mL aCE yielded unreliable data and was excluded from the study. A synergistic effect was observed at all other tested concentrations of aCE.

**Table 4:** Changes in the EC50 and corresponding *p*-values after the *C. cassia* aCE synergy experiment in the U937 cell line

aCE concentration	Infliximab EC50 [ng/mL]	*p*-values
Control	1.290	NA
50 µg/mL	0.9294	< 0.0001
25 µg/mL	0.938	0.0001
10 µg/mL	1.042	0.0087

As observed in the L929 assay, *C. zeylanicum* aCE showed significant synergy (*p* < 0.05) at 15 min and at all concentrations (Fig. 3B and Table 5). Synergistic effects were not observed for the two cinnamon species at any other time point or concentration. *C. loureiroi* aCE did not show synergy at any time point or concentration (data not shown).

**Table 5:** Changes in the EC50 and corresponding *p*-values after the *C. zeylanicum* aCE synergy experiment in the U937 cell line

aCE concentration	Infliximab EC50 [ng/mL]	*p*-values
Control	1.397	NA
50 µg/mL	1.125	0.0109
25 µg/mL	1.115	0.0001
10 µg/mL	1.138	0.0167

### Synergy experiments with purified cinnamon bioactives

**Fig. 4:**
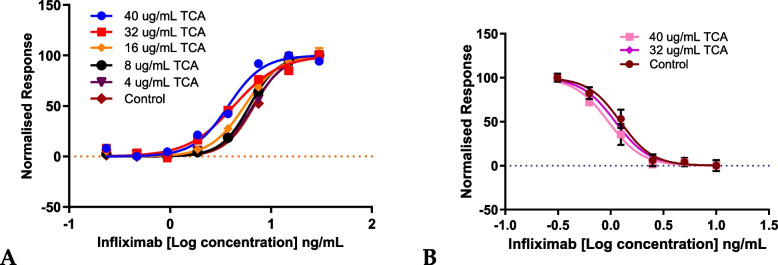
Synergy experiments with trans-cinnamaldehyde (TCA) at 15 min preincubation in the L929 (**A**) and U937 (**B**) cell lines. Dose-dependent synergy was observed with TCA. The *p*-values for TCA’s effect in the L929 (Table 6) and U937 cell lines (Table 7) have been summarized

TCA, CA, and eugenol are bioactive compounds found in cinnamon. To identify the component that contributed to the observed synergy, we included these three cinnamon bioactive compounds in our study. We determined the viability of L929 and U937 cells using Alamar Blue reagent and each of these bioactive compounds (data not shown), and the concentrations that were non-toxic to the cells were selected for the synergy experiments. We observed significant (*p* < 0.05) synergy between TCA and infliximab in L929 cells (Fig. 4A and Table 6).

**Table 6:** Changes in the EC50 and corresponding *p*-values after the trans-cinnamaldehyde (TCA) synergy experiment in the L929 cell line

*trans*-cinnamaldehyde concentration	Infliximab EC50 [ng/mL]	*p*-values
Control	6.800	NA
40 µg/mL	3.777	< 0.0001
32 µg/mL	4.275	< 0.0001
16 µg/mL	5.471	< 0.0001
8 µg/mL	6.124	0.0099
4 µg/mL	6.625	0.5145

In U937 cells, synergy between TCA and infliximab was observed at higher concentrations i.e., 40 and 32 µg/mL (Fig. 4B and Table 7). Surprisingly, none of the other purified compounds showed synergy in L929 or U937 cells.

**Table 7:** Changes in the EC50 and corresponding *p*-values after the TCA synergy experiment in the U937 cell line

Trans-cinnamaldehyde concentration	Infliximab EC50 [ng/mL]	*p*-values
Control	1.234	NA
40 µg/mL	0.9498	0.0007
32 µg/mL	0.9703	0.0008

### Synergy calculation and selection of cinnamon species for gene expression analysis

**Fig. 5:**
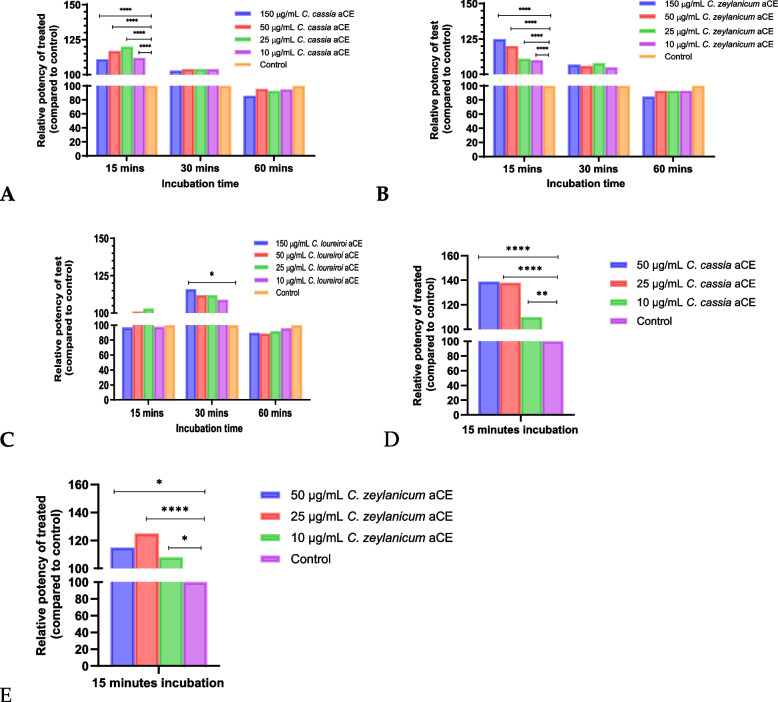
aCE increases the relative potency of infliximab in L929 and U937 cells. **A** Summary of the changes in infliximab relative potency after *C. cassia* aCE treatment at various time points in L929 cells. **B** Changes in infliximab relative potency after *C. zeylanicum* aCE treatment at various time points in L929 cells. **C** Changes in infliximab relative potency after *C. loureiroi* aCE treatment at various time points in L929 cells. **D** Changes in infliximab relative potency after *C. cassia* aCE treatment in U937 cells (**E**) Changes in infliximab relative potency after *C. zeylanicum* aCE treatment in U937 cells. *p* < 0.0005****, *p* < 0.01**, and *p* < 0.05*

The relative potency of infliximab with aCE and bioactive compounds was calculated by considering the ratio of infliximab’s EC50 without aCE/bioactive compound and that with aCE/bioactive compound and converting it to a percentage. The highest synergy in the L929 assay system resulted in 125% relative potency (150 µg/mL *C. zeylanicum* aCE) of infliximab with aCE against 100% without aCE (Fig. 5B).

**Fig. 6:**
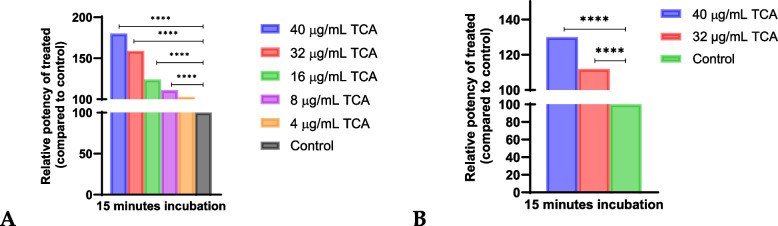
The cinnamon bioactive trans-cinnamaldehyde demonstrated significant dose-dependent synergy in the L929 (**A**) and U937 (**B**) cell lines. *p* < 0.0005****

Similarly, 120% and 110% relative potencies of infliximab were observed with 50 µg/mL *C. cassia* aCE (Fig. 5A) and 150 µg/mL *C. loureiroi* aCE, respectively (Fig. 5C) and 180% relative potency was observed with 40 µg/mL bioactive TCA (Fig. 6A).

The synergy was more pronounced in the U937 cells at 15 min, with the highest synergy resulting in 139% relative potency of infliximab with 50 µg/mL *C. cassia* aCE (Fig. 5D), 125% relative potency with 25 µg/mL *C. zeylanicum* (Fig. 5E) and 130% relative potency was observed with 40 µg/mL TCA(Fig. 6B).

Based on the data, we concluded that aCE and bioactive TCA work synergistically with infliximab. Next, we aimed to identify the genes that were critical in infliximab non-responders and the pathways that could be examined to further understand the role of aCE and bioactives in the observed synergy with infliximab. We selected genes involved in critical signaling pathways [Janus kinase 2 (JAK2), signal transducer and activator of transcription 3 (STAT3), and myeloid differentiation factor 88 (MyD88)], apoptosis [B-cell lymphoma 2 (Bcl-2), B-cell lymphoma-extra-large (Bcl-xL), and Bcl-2-associated X protein (BAX)], adhesion [intercellular adhesion molecule-1 (ICAM-1) and vascular cell adhesion molecule-1 (VCAM-1)], and innate immune response [Toll-like receptor-2 (TLR-2) and TLR-4] and those encoding matrix metalloproteinases and tissue inhibitor of metalloproteinases [matrix metalloproteinase 1 (MMP1), MMP3, and tissue inhibitor of metalloproteinases (TIMP-1)].

### Gene expression analysis

From published studies, we identified genes, the expression of which was elevated in infliximab non-responders and were either directly involved in disease pathogenesis or were therapeutic indicators of non-response. We selected *C. cassia, C. zeylanicum*, and TCA for the gene expression analysis based on their performance in the synergy experiments. Two concentrations each of aCE (50 and 25 µg/mL) and TCA (40 and 32 µg/mL) were used for gene expression analysis in both L929 and U937 cell lines.

Cytochrome C1 (*CYC1*) and beta-2-microglobulin (*B2M*) were used as housekeeping genes in L929 cells. Ribosomal protein lateral stalk subunit P2 (*RPLP2*) and glyceraldehyde 3-phosphate dehydrogenase (*GAPDH*) were used as housekeeping genes for the U937 cell line. Gene expression analysis was performed Normfinder. These genes were selected from the six-gene panel (for each cell line) because the Ct values obtained across different treatments did not vary much across treated, untreated and control samples (data not shown).

### Changes in the expression of adhesion molecules (ICAM-1 and VCAM-1) after various treatments

**Fig. 7:**
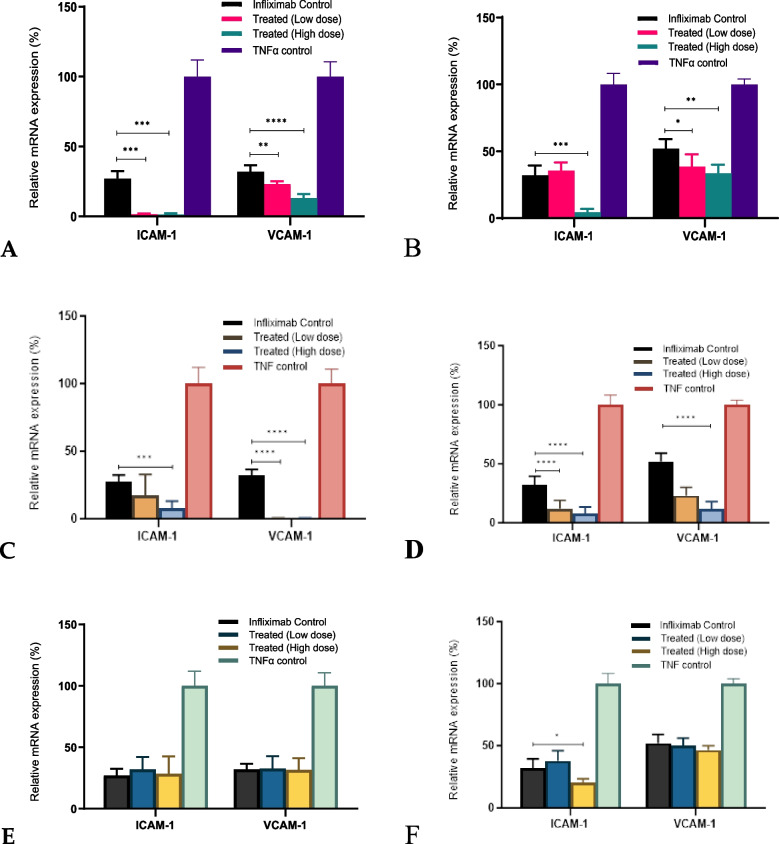
High (50 µg/mL) and low (25 µg/mL) concentrations of *C. cassia* aCE (**A** (L929) and **B** (U937)) and high (40 µg/mL) and low (32 µg/mL) concentrations TCA (**C** (L929) and **D** (U937)) downregulated the mRNA levels of *ICAM-1* and *VCAM-1* synergistically with infliximab. *C. zeylanicum* aCE did not show any effect on the expression of the selected mRNAs (**E** (L929) and **F** (U937))

We observed an increase in the expression of *ICAM-1* and *VCAM-1* with TNF-α treatment, which was reversed with infliximab treatment. Next, we aimed to determine whether aCE or TCA could act synergistically to improve the effect of infliximab**.** *C. cassia* aCE and TCA worked synergistically with infliximab and downregulated the mRNA levels of *ICAM-1* and *VCAM-1* in both cell lines. Higher doses of *C. cassia* aCE (50 µg/mL) reduced the mRNA expression of *ICAM-1* by an additional ~ 25.6 − 27.7% and that of *VCAM-1* by ~ 19% (Figs. 7A (L929) and B (U937)). TCA (40 µg/mL) downregulated *ICAM-1* mRNA expression by an additional ~ 18.9 − 24.1% and that of *VCAM-1* by ~ 31.9 − 41% (Figs. 7C (L929) and D (U937)). *C. zeylanicum* did not show any synergistic effects (Figs. 7E (L929) and F (U937)) in either cell line.

### Changes in the expression of innate immune response receptors (TLR-2 and TLR-4) after various treatments

**Fig. 8:**
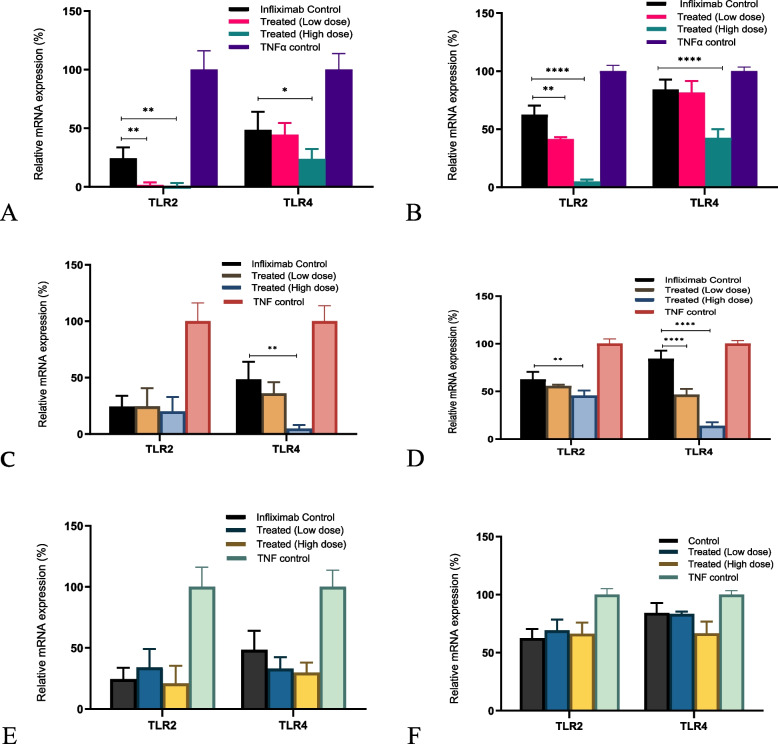
*C. cassia* aCE (**A** (L929) and **B** (U937)) was better at downregulating the expression of both Toll-like receptors than TCA (**C** (L929) and **D** (U937)), TCA downregulated the expression of TLR-4 more than TLR-2. Synergistic effects were more pronounced in U937 (**B** and **D**) than in L929 (**A** and **C**). *C. zeylanicum* aCE did not affect the expression of the selected mRNAs (**E** (L929) and **F** (U937))

*TLR-2* and *TLR-4* mRNA expression increased in the presence of TNF-α and decreased in the presence of infliximab. This decrease was more pronounced in the U937 (Fig. 8 B and D) cell line than in the L929 cells (Fig. 8A and C). *C. cassia* aCE was better at downregulating the expression of both Toll-like receptors than TCA (Fig. 8C and D), and downregulated *TLR-2* more than *TLR-4.* Higher doses of *C. cassia* aCE (50 µg/mL) reduced the mRNA expression by an additional ~ 23.6 − 57.7% for *TLR-2*, and by 24.7–41.7% for *TLR-4* (Fig. 8A (L929) and B (U937)). TCA (40 g/mL) downregulated *TLR-2* mRNA expression by an additional 4–17.1% and *TLR-4* expression by ~ 43.8–73.2% (Fig. 8C (L929) and D (U937)). *C. zeylanicum* did not exhibit any synergistic effects (Fig. 8E (L929) and F (U937)).

### Changes in the expression of matrix metalloproteinases and TIMP-1 after various treatments

**Fig. 9:**
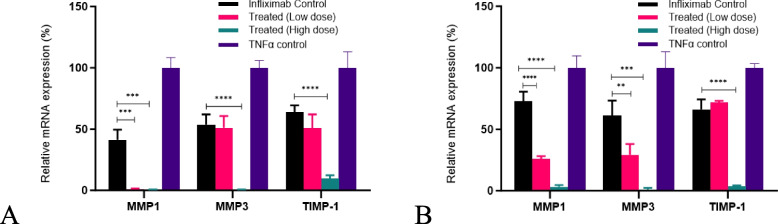
*C. cassia* aCE was the only extract that showed synergistic activity with infliximab in downregulating *MMP1, MMP3,* and *TIMP-1* mRNAs (**A** (L929) and **B** (U937))

We observed an increase in the mRNA levels of *MMP1, MMP3*, and *TIMP-1* upon TNF-α treatment. This increase was partially reversed by infliximab treatment (Figs. 9A (L929) and B (U937)). Higher doses of *C. cassia* aCE (50 µg/mL) decreased *MMP1* expression by an additional ~ 40.9 − 69.5%, *MMP3* expression by ~ 53.3 − 60.4%, and *TIMP-1* expression by ~ 54.3 − 62.9%. The other test samples (*C. zeylanicum* and TCA) did not show any synergistic effects with infliximab in either of the cell lines (data not shown).

#### Changes in apoptosis-related (Bcl-2, Bcl-xL, and BAX) gene expression after various treatments

**Fig. 10:**
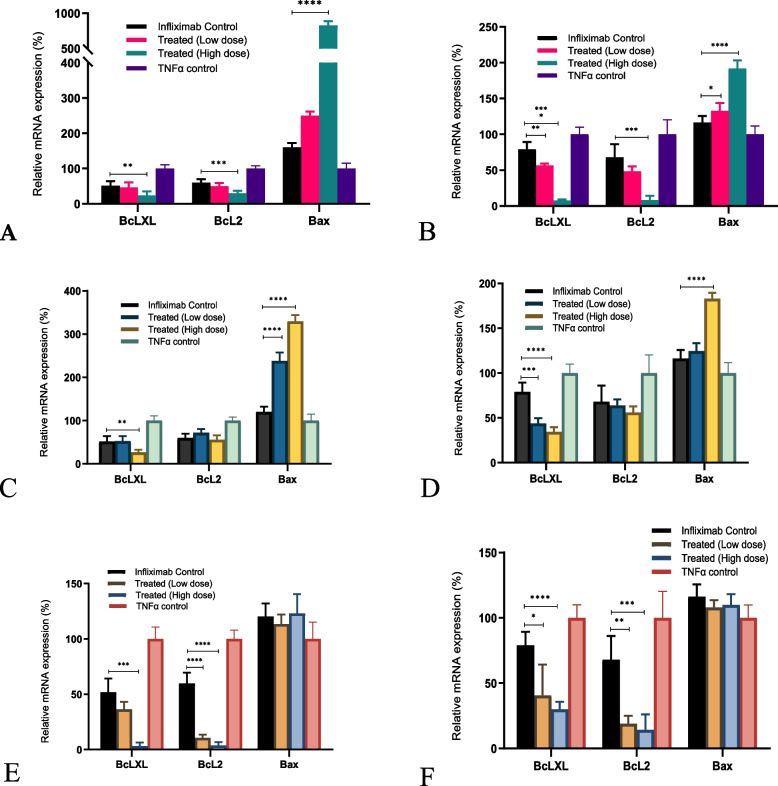
TCA decreased the expression of *Bcl-xL* and *Bcl-2* mRNAs. Downregulation was observed with both cell lines. *C. cassia* aCE in L929 (**A**) and U937 (**B**) and *C. zeylanicum* aCE in the L929 (**C**) and U937 cell lines (**D**). TCA was also effective in downregulating the *Bcl-xL* and *Bcl-2* mRNAs with an additive effect (Fig. 10 **E** (L929) and **F** (U937)). The pro-apoptotic *BAX* mRNA was upregulated in the presence of infliximab and aCE (**A**, **B**, **C**, and **D**). TCA, however, did not affect *BAX* expression (**E** and **F**)

Bcl-xL and Bcl-2 expression increased after TNF-α treatment, which was downregulated in the presence of infliximab. *C. cassia aCE* and the bioactive TCA synergistically downregulated Bcl-xL and Bcl-2 expression. Higher doses of *C. cassia* aCE (50 µg/mL) decreased the expression of Bcl-xL by an additional ~ 28.0–71.6% (Fig. 10A (L929) and B (U937)), while TCA (40 µg/mL) reduced it by ~ 38.4–48.6% (Fig. 10E (L929)and F (U937)). *C. zeylanicum* (50 µg/mL) downregulated Bcl-xL by ~ 25.0–44.9% but did not affect Bcl-2 expression (Fig. 10C (L929) and D (U937)). Bcl-2 expression was downregulated by an additional ~ 29.7–60.7% by 50 µg/mL *C. cassia* aCE (Fig. 10A (L929) and B (U937)) and by ~ 53.8–56.1% by 40 µg/mL TCA (Fig. 10E (L929) and F (U937)). *BAX,* a pro-apoptotic gene, was additionally upregulated in the presence of aCE (50 µg/mL) (Fig. 10A, B, C, and D). A ~ 16.5–708.3% increase in *BAX* mRNA was observed with *C. cassia* aCE (Fig. 10A (L929) and B (U937)), whereas the extent of upregulation was lower (~ 66.6 – 209.8%) with *C. zeylanicum* aCE (Fig. 10C (L929) and D (U937)) and TCA did not affect *BAX* expression (Fig. 10E (L929) and F (U937)).

#### Changes in the expression of transcription factors (MyD88, JAK2, and STAT3) after various treatments

**Fig. 11:**
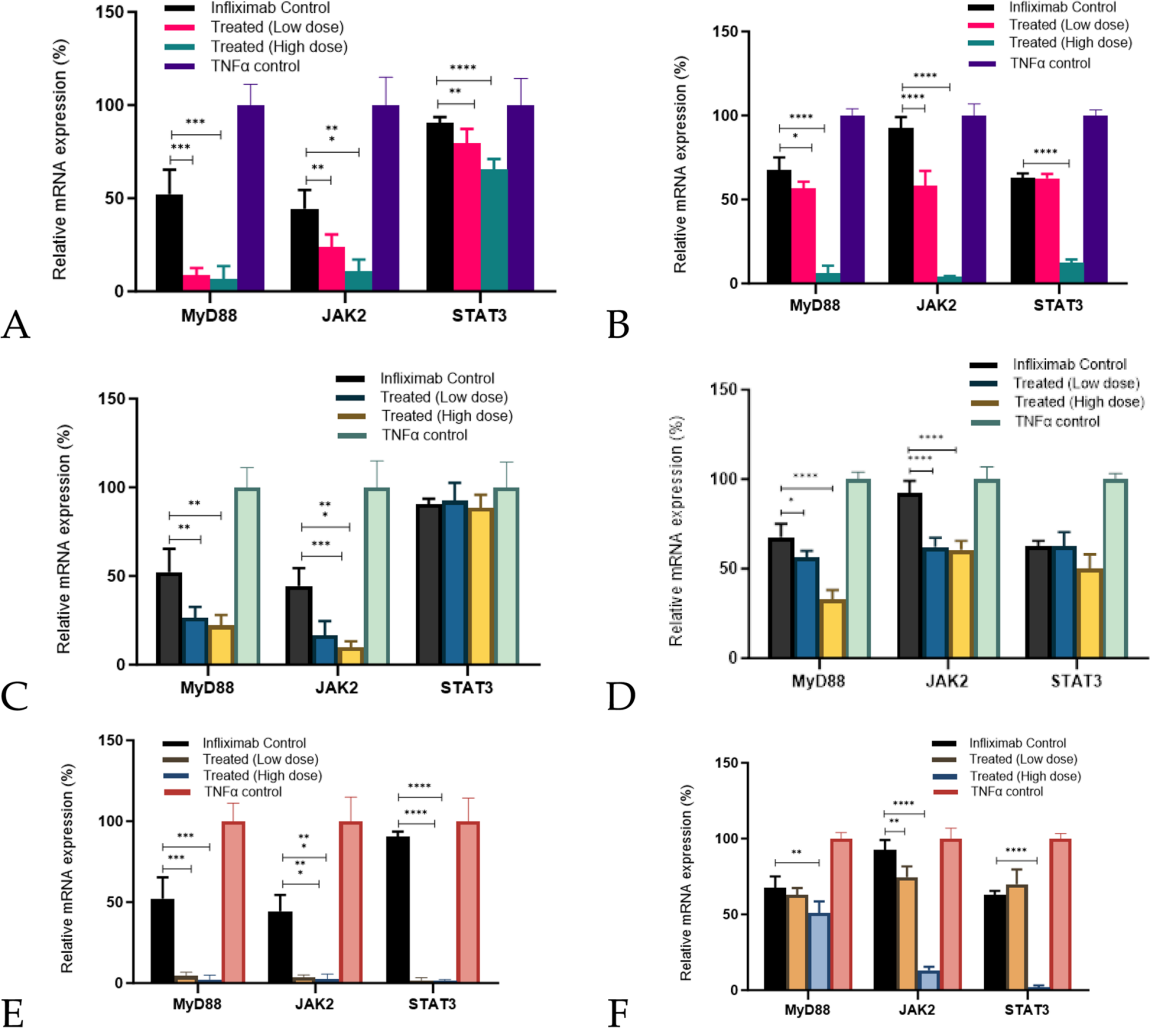
Downregulation of MyD88 and JAK2 was observed with both *C. cassia* (**A** (L929) and **B** (U937)) and *C. zeylanicum* aCE (**C** (L929) and **D** (U937)). *C. cassia* aCE was effective in downregulating *STAT3* (A (L929) and B (U937)) synergistically, although *C. zeylanicum* did not affect *STAT3* mRNA expression (**C** (L929) and **D** (U937)). TCA decreased *MyD88, JAK2*, and *STAT3* mRNA levels synergistically in both cell lines (**E** (L929) and **F** (U937))

Transcription factors such as MyD88, JAK2, and STAT3 are critical regulators of several signaling pathways such as for TLRs (MyD88), IL-6 and IL12 (JAK2 and STAT3) which play a key role in IBD [24, 25]. Hence, we assessed the synergistic effects of aCE and infliximab on the expression of these transcription factors. The *C. cassia* aCE synergistically downregulated MyD88 by ~ 45.8–61.8% (Fig. 11A (L929) and B (U937)), while TCA downregulated it by ~ 16.5–50.2% (Fig. 11E (L929) and F (U937)). *STAT3* was downregulated 25.3–50.4% by *C. cassia* aCE (Fig. 11A (L929) and B (U937)) and by ~ 60.9–89.2% by TCA (Fig. 11E (L929) and F (U937)). JAK2 was downregulated ~ 33.4–88.3% by *C. cassia* aCE (Fig. 11A (L929) and B (U937)) and by ~ 42–79.4% by TCA (Figs. 11E (L929) and F (U937)). The *C. zeylanicum* aCE did not show any additive effect on STAT3 levels. For other transcription factors, an additive effect was observed: ~ 30–34.7% for MyD88 and ~ 31.8–34.5% for TCA (Figs. 11C (L929) and D (U937)).

## Discussion

In this study, we investigated whether CAM could act synergistically in infliximab non-response situations. Several herbal products have been reported to help manage chronic inflammatory conditions, such as IBD and RA [20, 21]. We selected cinnamon, which is known to possess several beneficial properties, including anti-inflammatory activity [26]. We studied the synergistic effects of cinnamon and infliximab in vitro using a two-step approach. First, we investigated the effect of aCE pretreatment on infliximab potency. The second phase involved the selection of conditions that supported statistically significant synergy with infliximab and the study of the mRNA expression of several genes that are reported to be critical in non-response situations to infliximab under significant synergistic conditions with aCE.

We tested the aCE of three cinnamon species: *C. cassia*, *C. zeylanicum, and C. loureiroi*. These species vary in their chemical composition and contribute differently to various testing conditions and indications [27, 28]. We also assessed the species-specific effects. We observed significant synergy between infliximab and the aCEs of *C. cassia* and *C. zeylanicum*. Surprisingly, we did not observe synergy between the biotherapeutic and the aCE of *C. loureiroi* in phase one of our study. We tested the synergy of several bioactive cinnamon compounds (TCA, CA, and eugenol) and observed that only TCA showed significant synergy with infliximab.

A synergistic effect was observed in two cell lines, L929 (a mouse fibroblast cell line) and U937 (a human monocyte cell line). In our assay, we preincubated the cells with aCE for different durations. The best experimental condition included preincubation for 15 min, followed by the addition of infliximab. A dose-dependent increase in synergy was observed from to 10 − 50 µg/mL aCE, as was evident from the reduction in the EC50 of infliximab. The data pertaining to 150 µg/mL aCE varied between *C. cassia* and *C. zeylanicum*. In terms of relative potency, we observed a 125% increase in the potency of infliximab in the presence of aCE and 180% in presence of TCA. Infliximab alters the expression of adhesion molecules, pro-apoptotic genes, and transcription factors [29]. Additionally, some studies have shown that TLRs and MMPs contribute to non-response [30, 31], the expression of which is modulated by infliximab. Cinnamon extract and its bioactive compounds possess anti-inflammatory properties and downregulate the expression of TLR, MyD88 [24], JAK/STAT [32], Bcl/BAX [33], ICAM-1, VCAM-1 [34], and MMPs [35]. We investigated the additive effects of infliximab and aCE and its bioactives on the expression of adhesion molecules (ICAM-1 and VCAM-1), innate immune response receptors (TLR-2 and TLR-4), MMPs and TIMP (MMP1, MMP3, and TIMP-1), apoptosis-related genes (Bcl-2, BcL-xL, and BAX), and transcription factors (JAK2, STAT3, and MyD88). We selected 25 and 50 µg/mL aCE and 32 and 40 µg/mL bioactive TCA for our phase two experiments to study the synergy between infliximab and aCE at the molecular level.

In a recent study, TLR-2 was identified as one of the markers of a non-response pathway [31]. In another study, it was reported that infliximab decreased the expression of TLR-4 and TLR-5, the study indicated that higher TLR-4 and TLR-5 expression exacerbated inflammatory conditions in ankylosing spondylitis [36]. TLRs are pattern recognition receptors that are essential components of the innate immune system. TLR signal transduction is mediated via the MyD88-dependent and independent pathways, eventually activating NF-κB and releasing various pro-inflammatory cytokines [24]. In our study, we observed that aCE synergistically downregulated the expression of *TLR-2* and *TLR-4* in the presence of infliximab. However, only *C. cassia* and TCA showed synergy, and the results in the monocytic U937 cell line were more pronounced than those in the L929 fibroblasts. The *C. zeylanicum* extract did not exhibit any synergistic effect in either cell line. Both *C. cassia* and *C. zeylanicum* extracts and TCA synergistically decreased *MyD88* mRNA levels.

The pathogenesis of IBD is not entirely understood, and reports have indicated that a combination of factors is involved in disease manifestation, including the gut immune homeostasis. Environmental factors and genetic predispositions disrupt the balance between immune cells and intestinal microbes and contribute to an inflammatory environment. Cell adhesion molecules, such as ICAM-1 (required for leukocyte adhesion) and VCAM-1 (required for monocyte-endothelial cell interaction), along with other adhesion molecules, are involved in maintaining gut homeostasis, and their expression is elevated under inflammatory conditions [37, 38]. In active IBD, leukocytes and macrophages are recruited into the affected mucosa, which results in the production of a milieu of pro-inflammatory cytokines such as TNF-α, interferon (IFN)-γ, interleukin (IL)-6, and IL-1β. Infliximab treatment results in TNF-α neutralization, thereby decreasing the levels of various pro-inflammatory cytokines, including that of TNF-α. Reports indicate that infliximab downregulates *ICAM-1* and *VCAM-1* mRNA levels in phytohemagglutinin and lipopolysaccharide-stimulated human peripheral blood leukocytes and U937 cells [39]. Yarur et al. [40] have shown that the levels of VCAM-1 and IL-6 were significantly higher in patients that were exposed to multiple anti-TNF-α agents. This observation is significant, as it shows that simply switching the anti-TNF-α agent in non-responders may not be beneficial. Therefore, new approaches should be identified. Our study showed that aCE from *C. cassia* and the bioactive TCA significantly decreased the mRNA expression of *ICAM-1* and *VCAM-1* synergistically with infliximab in both cell lines.

MMPs are also upregulated under inflammatory conditions and have been reported to influence gut homeostasis. MMPs are involved in the degradation of the extracellular matrix, cytokines, and adhesion molecules. TIMP neutralizes MMPs, and a balance between TIMP and MMP expression maintains MMP activity [41]. Several MMPs, such as MMP1, 2, 3, and 7 are upregulated in IBD [42], which alters the MMP-TIMP ratio and further contributes to pathogenesis. Furthermore, in some patients, MMP3 cleaved IgG, thereby affecting the bioavailability of the anti-TNF-α agents and contributing to the lack of response [43]. We tested the effects of aCE on the expression of *MMP1, MMP3*, and *TIMP-1*. Only *C. cassia* aCE synergistically decreased the expression of MMPs and *TIMP-1* in both cell lines, albeit only at higher concentrations. Klimuk et al. [44] have reported that initial infliximab infusion significantly reduces MMP1, 3, and 9 and TIMP-1 and 2 expressions. Our observations are in agreement with published data.

With increasing knowledge regarding the pathobiology of IMIDs, multiple cytokines and receptors have been identified as targets for therapy, which include members of the cytokine type 1 and 2 families, the transforming growth factor-β family, and TNF-α and its receptors [45]. The cytokine, IL-6, plays an important role in mediating host defense in response to tissue injury or infection, and its expression is controlled stringently. An aberrant increase in IL-6 level exacerbates inflammatory conditions [46]. IL-6 binds to and activates the JAK2-STAT3 signaling pathway. Recently, inhibitors of the JAK-STAT pathway, such as tofacitinib, have been found to be effective against IBIDs [25]. However, adverse events associated with tofacitinib such as pulmonary embolism and herpes zoster infection have been reported. Furthermore, this inhibitor is not equally effective against different IMIDs [47].

Over the last decade, the effects of phytochemicals on inflammation have been investigated extensively and the results are encouraging. Moon et al. [48] have summarized the effects of a few phytochemicals, such as phenolics, terpenoids, nitrogen-containing alkaloids, and sulfur-containing compounds, which are effective in managing IMIDs. Interestingly, these compounds also affected the JAK-STAT pathway. We demonstrated that *C. cassia* aCE and TCA, when supplemented with infliximab, reduced the mRNA levels of *JAK2* and *STAT3* in a dose-dependent manner. *C. zeylanicum* aCE does not affect STAT3 expression. In addition to JAK-STAT, we checked the mRNA levels of the downstream anti-apoptotic genes, *Bcl-2* and *Bcl-xL*, and the pro-apoptotic gene, *BAX*. We observed a decrease in *Bcl-2* and *Bcl-xL* mRNA levels, whereas *BAX* mRNA levels were significantly and synergistically elevated. The expression of the *Bcl-2* family of genes is mediated via the JAK-STAT pathway [49], and the ratio of Bax to Bcl-2 has been reported to be a predictor of infliximab response. An increase in the ratio of Bax to Bcl-2 increases apoptosis, which is mediated by increased caspase 3 expression [50]. *C. cassia* aCE showed a dose-dependent effect on all the selected apoptotic genes, whereas *C. zeylanicum* aCE did not affect *Bcl-2*, and TCA did not affect the *BAX* mRNA level.

Our study has certain limitations. The primary limitation was performing only in vitro experiments. In vivo studies will provide more conclusive data and corroborate our in vitro findings. Additionally, we did not test the effects of gene polymorphisms on aCE treatment. Many genetic markers (*IL-6, TNFRSF1A, TLR-2*, and *JAK2*) are associated with non-response [51], and an aCE-based study on polymorphic variants for critical cytokines/receptors will be beneficial in the future.

In conclusion, we have shown that cinnamon works synergistically with the targeted biotherapeutic, infliximab, when used either as an extract or as a specific bioactive agent to manage inflammation. Our data were obtained using aCE, which is easy to prepare and can be used for in vivo testing. We observed species-specific differences in the tested parameters, and *C. cassia* aCE and TCA were found to be more effective in inducing the additive effect. The *C. zeylanicum* aCE was effective for some of the tested parameters. Targeting of cytokines and their signaling has been a successful approach, although it is associated with non-response in many patients (~ 40 − 50%). Furthermore, in IMIDs, a plethora of cytokines are responsible for the pathological conditions and targeting multiple cytokines/receptors simultaneously may improve the rate of remission [47]. Overall, this study paves the way for the development of a synergistic approach for treating IMIDs. Many natural products possess anti-inflammatory properties and can be utilized as CAMs to improve the quality of life of patients. Natural products can be used as a standalone treatment, where they have been proven to be safe and efficacious [20], or as a supplement [23]. Our study provides evidence that aCE exerts a species-specific synergistic effect with infliximab. In vivo studies are required to investigate whether aCE of various species can be used as a CAM to improve patients’ quality of life.

### Supplementary Information

**Supplementary Material 1.**

## Data Availability

All data generated or analyzed during this study are included in this published article. Contact the corresponding author to request data from this study.
