# Supplementary material for: An in vitro study elucidating the synergistic effects of aqueous cinnamon extract and an anti-TNF-α biotherapeutic: implications for a complementary and alternative therapy for non-responders
Source: BMC Complement Med Ther. 2024 Mar 23;24:131. doi: 10.1186/s12906-024-04438-w (PMC10960381; doi:10.1186/s12906-024-04438-w)
Supplement: Supplementary file 1 — Supplementary Material 1. [file 12906_2024_4438_MOESM1_ESM.pdf]

	All primer sequences 5' to 3', for U937 cell extracts
Human RPLP2	(F) TCTTGGACAGCGTGGGTATCGA
	(R) CAGCAGGTACACTGGCAAGCTT
Human GAPDH	(F) GTCTCCTCTGACTTCAACAGCG
	(R) ACCACCCTGTTGCTGTAGCCAA
Human TLR2	(F) CTTCACTCAGGAGCAGCAAGCA
	(R) ACACCAGTGCTGTCCTGTGACA
Human TLR4	(F) CCCTGAGGCATTTAGGCAGCTA
	(R) AGGTAGAGAGGTGGCTTAGGCT
Human VCAM-1	(F) GATTCTGTGCCCACAGTAAGGC
	(R) TGGTCACAGAGCCACCTTCTTG
Human ICAM-1	(F) AGCGGCTGACGTGTGCAGTAAT
	(R) TCTGAGACCTCTGGCTTCGTCA
Human TIMP-1	(F) GGAGAGTGTCTGCGGATACTTC
	(R) GCAGGTAGTGATGTGCAAGAGTC
Human MMP 1	(F) ATGAAGCAGCCCAGATGTGGAG
	(R) TGGTCCACATCTGCTCTTGGCA
Human MMP 2	(F) CTGAAGGTGATGAAGCAGCC
	(R) AGTCCAAGAGAATGGCCGAG
Human STAT 3	(F) CTTTGAGACCGAGGTGTATCACC
	(R) GGTCAGCATGTTGTACCACAGG
Human JAK 2	(F) CCAGATGGAAACTGTTCGCTCAG
	(R) GAGGTTGGTACATCAGAAACACC
Human MyD88	(F) GAGGCTGAGAAGCCTTTACAGG
	(R) GCAGATGAAGGCATCGAAACGC
Human Bcl2	(F) ATCGCCCTGTGGATGACTGAGT
	(R) GCCAGGAGAAATCAAACAGAGGC
Mouse Bcl xl	(F) GCCACCTATCTGAATGACCACC
	(R) AGGAACCAGCGGTTGAAGCGC
Human Bax	(F) TCAGGATGCGTCCACCAAGAAG
	(R) TGTGTCCACGGCGGCAATCATC

	All primer sequences 5' to 3' - For L929 cell extracts
CYC1 Mouse	(F) CCATCTACACAGAAGTCTTGGAG
	(R) GCGTTTTCGATGGTCATGCTCTG
β2M Mouse	(F) ACAGTTCCACCCGCCTCACATT
	(R) TAGAAAGACCAGTCCTTGCTGAAG
Mouse TLR2	(F) ACAGCAAGGTCTTCCTGGTTCC
	(R) GCTCCCTTACAGGCTGAGTTCT
Mouse TLR4	(F) AGCTTCTCCAATTTTTCAGAACTTC
	(R) TGAGAGGTGGTGTAAGCCATGC
Mouse VCAM-1	(F) GCTATGAGGATGGAAGACTCTGG
	(R) ACTTGTGCAGCCACCTGAGATC
Mouse ICAM-1	(F) AAACCAGACCCTGGAACTGCAC
	(R) GCCTGGCATTTCAGAGTCTGCT
Mouse TIMP-1	(F) TCTTGGTTCCCTGGCGTACTCT
	(R) GTGAGTGTCACTCTCCAGTTTGC
Mouse MMP 1	(F) AGGAAGGCGATATTGTGCTCTCC
	(R) TGGCTGGAAAGTGTGAGCAAGC
Mouse MMP 2	(F) CAAGGATGGACTCCTGGCACAT
	(R) TACTCGCCATCAGCGTTCCCAT
Mouse STAT 3	(F) AGGAGTCTAACAACGGCAGCCT
	(R) GTGGTACACCTCAGTCTCGAAG
Mouse JAK 2	(F) GCTACCAGATGGAAACTGTGCG
	(R) GCCTCTGTAATGTTGGTGAGATC
Mouse MyD88	(F) ACCTGTGTCTGGTCCATTGCCA
	(R) GCTGAGTGCAAACTTGGTCTGG
Mouse Bcl2	(F) CCTGTGGATGACTGAGTACCTG
	(R) AGCCAGGAGAAATCAAACAGAGG
Mouse Bcl xl	(F) GCCACCTATCTGAATGACCACC
	(R) AGGAACCAGCGGTTGAAGCGC
Mouse Bax	(F) AGGATGCGTCCACCAAGAAGCT
	(R) TCCGTGTCCACGTCAGCAATCA
